# OnfD, an AraC-Type Transcriptional Regulator Encoded by *Rhizobium tropici* CIAT 899 and Involved in Nod Factor Synthesis and Symbiosis

**DOI:** 10.1128/AEM.01297-20

**Published:** 2020-09-17

**Authors:** Pablo del Cerro, Paula Ayala-García, Pablo Buzón, Roger Castells-Graells, Francisco Javier López-Baena, Francisco Javier Ollero, Francisco Pérez-Montaño

**Affiliations:** a Departamento de Microbiología, Facultad de Biología, Universidad de Sevilla, Seville, Spain; b Department of Biological Chemistry, John Innes Centre, Norwich Research Park, Norwich, United Kingdom; University of Illinois at Chicago

**Keywords:** AraC-type regulators, *Rhizobium tropici*, legumes, plant-microbe interactions, regulation of gene expression, symbiosis, transcriptional regulation

## Abstract

Rhizobium tropici CIAT 899 is a broad-host-range rhizobial strain that establishes symbiotic interactions with legumes and tolerates different environmental stresses such as heat, acidity, or salinity. This rhizobial strain produces a wide variety of symbiotically active nodulation factors (NF) induced not only by the presence of plant-released flavonoids but also under osmotic stress conditions through the LysR-type transcriptional regulators NodD1 (flavonoids) and NodD2 (osmotic stress). However, the activation of NodD2 under high-osmotic-stress conditions remains elusive. Here, we have studied the role of a new AraC-type regulator (named as OnfD) in the symbiotic interaction of *R. tropici* CIAT 899 with Phaseolus vulgaris and *Lotus* plants. We determined that OnfD is required under salt stress conditions for the transcriptional activation of the nodulation genes and therefore the synthesis and export of NF, which are required for a successful symbiosis with P. vulgaris. Moreover, using bacterial two-hybrid analysis, we demonstrated that the OnfD and NodD2 proteins form homodimers and OnfD/NodD2 form heterodimers, which could be involved in the production of NF in the presence of osmotic stress conditions since both regulators are required for NF synthesis in the presence of salt. A structural model of OnfD is presented and discussed.

**IMPORTANCE** The synthesis and export of rhizobial NF are mediated by a conserved group of LysR-type regulators, the NodD proteins. Here, we have demonstrated that a non-LysR-type regulator, an AraC-type protein, is required for the transcriptional activation of symbiotic genes and for the synthesis of symbiotically active NF under salt stress conditions.

## Abstract (precis)

The synthesis and export of rhizobial NF are mediated by a conserved group of LysR-type regulators, the NodD proteins. Here, we have demonstrated that a non-LysR-type regulator, an AraC-type protein, is required for the transcriptional activation of symbiotic genes and for the synthesis of symbiotically active NF under salt stress conditions.

## INTRODUCTION

Rhizobia are soil proteobacteria that establish symbiosis with legumes. As an outcome of this mutualistic interaction, plants develop nodules, root organs where bacteria differentiate into bacteroids. This differentiated endosymbiotic form reduces atmospheric nitrogen to ammonia, increasing the availability of this constraining nutrient for the plant (1). The rhizobium-legume symbiosis requires a complex and evolved molecular exchange of signals between both partners of the symbiosis. This molecular dialogue is initiated by the legume roots' exudation of flavanoids, phenylpropanoid metabolites that are recognized by bacterial NodD proteins. These proteins bind to conserved promoter regions located upstream of nodulation (*nod*) genes, the *nod* boxes (NB), activating their expression (2–4). The *nod* genes are involved in the synthesis and export of specific lipochitooligosaccharides or Nod factors (NF) that, in turn, trigger different plant processes essential for nodulation success, such as root hair deformation, the development of the infection thread, and the formation of root nodule primordia (5–7). In addition to NF, other molecules and/or processes are also important for a successful legume-rhizobium symbiosis, such as surface polysaccharides, phytohormone production, motility, quorum sensing, and protein secretion systems (8–14).

Rhizobium tropici CIAT 899 is a broad-host-range rhizobial strain isolated from tropical acidic soils of South America that is tolerant to diverse environmental stresses, such as heat, acidity, or salinity, and induces the formation of nitrogen-fixing nodules on several legumes, including Phaseolus vulgaris, Lotus japonicus, and Lotus burttii (15–17). The availability of a CIAT 899 genome and transcriptome public database, the strain's tolerance to multiple abiotic stresses, and the use this rhizobium as an inoculant for plant crops have made CIAT 899 a model strain in the rhizobium-legume symbiosis field (12, 18–22). The CIAT 899 genome has multiple copies of *nodD* (*n* = 5) and *nodA* (*n* = 3) genes in the symbiotic plasmid (20). Previous reports assessed the role of the five NodD and the three CIAT 899 NodA proteins in the symbiotic performance with the above-mentioned legume hosts and found that *nodD1* or both *nodA1* and *nodA3* genes are required for the formation of nitrogen-fixing nodules in *L. japonicus* but not in *L. burttii* and P. vulgaris. In the common bean, the mutation of both *nodD1* and *nodD2* or of the three *nodA* genes simultaneously blocks the nodulation process (21, 23). Nevertheless, the absence of NodD2 or simultaneously of NodA1 and NodA3 has detrimental effects on the symbiotic performance with P. vulgaris.

The presence of inducing flavonoids (apigenin) and also osmotic stress conditions (salt or mannitol) induces the production of a wide variety of symbiotically active NF in CIAT 899. The synthesis of these NF is regulated by NodD1 (apigenin) and NodD2 (osmotic stress) (15, 16, 21, 22). Our previous transcriptomic study showed that most of the CIAT 899 symbiosis-related genes (*nodA1BCSUIJH* [controlled by *nod* box 1, NB1], *nodA2 hsnT nodFE* [NB2], *nodM* [NB3], *y4wEF* [NB4], and two genes with unknown functions, *rtciat899_pb01550* and *rtciat899_pb01545* [NB5]) are upregulated in the presence of both inducing molecules (12). However, a set of genes located downstream of NB9, *nodD2* and RTCIAT899_PB01075 (coding for an AraC-type transcriptional regulator), are only activated under salt stress (12). Intriguingly, even though NodD1 is not directly involved in the activation of most symbiotic genes under osmotic stress conditions, the upregulation of these two genes depends on the presence of this protein (21). This finding suggests that NodD1 could be enhancing *nodD2* expression, but it is still unknown how salt induces the activation of the NodD2 protein.

The first described *araC* gene, in Escherichia coli, codes for a transcriptional regulator that controls the transcription of genes required for the uptake and catabolism of the sugar l-arabinose, the *araBAD* operon. AraC is sensitive to the level of arabinose, playing a dual role in controlling the expression of the *araBAD* and *araC* genes via the P_araBAD_ and P_araC_ promoters, respectively. The AraC protein is an activator of these genes in the presence of arabinose but acts as a repressor in the absence of this sugar (24). Regulation of both promoters by the AraC protein has been extensively characterized (25). In the absence of arabinose, one monomer of the AraC dimer occupies the araI1 (arabinose inducer site I) site, while the other occupies a site ∼200 bp upstream, known as araO2 (arabinose operator site I) (26–28), leading to the formation of a DNA loop that blocks RNA polymerase from binding to the operon promoter (29, 30). However, when arabinose binds to the dimeric AraC, the AraC protein undergoes a conformational change and occupies the adjacent inducer sites araI1 and araI2, resulting in the induction of the P_araBAD_ and P_araC_ promoters (31, 32). Therefore, arabinose destabilizes the AraC protein's binding to the araI1-araO2 looped complex but stabilizes the protein by binding to the araI1-araI2 sites. Thousands of AraC-type members have been identified, and some of them have been characterized in the *Bacteria* domain. These proteins are involved in the transcriptional regulation of a variety of important cellular processes, including carbon metabolism, activation of protein secretion systems, virulence, and abiotic stress responses (33–37). In comparison, description and functioning of AraC-like regulator proteins have been poorly investigated in rhizobia. In Sinorhizobium meliloti 2011, an AraC-like transcription factor (CuxR) stimulates exopolysaccharide biosynthesis at elevated c-di-GMP levels (38), and it is also required for the induction of genes needed for α-galactoside utilization (39). In S. meliloti 2011, genes involved in the biosynthesis and transport of a siderophore produced under iron-starving conditions, the *rhbABCDEF* operon, are positively regulated by the product of the *rhrA* gene, an AraC-type transcriptional activator (40). In *Bradyrhizobium* sp. strain HW13, the AraC-type transcriptional regulator CadR controls the expression of the *cadABKC* cluster, which is involved in the conversion of several phenoxyacetic acids to their corresponding phenol derivatives (41). To our knowledge, there is only one report of an AraC-type protein involved in symbiosis in *S. fredii* NGR234. This bacterium improves its ability to symbiose with P. vulgaris through the heterologous expression of *orf816* (coding for an AraC/XylS-type transcriptional regulator) from *Rhizobium* sp. strain BR816 (42).

We have studied here the role in symbiosis of an undescribed AraC-type transcriptional regulator encoded by the *R. tropici* CIAT 899 RTCIAT899_PB01075 gene. We determined that this AraC-type regulator is necessary for a successful symbiosis with P. vulgaris and is involved in the transcriptional activation of the *nod* genes and NF synthesis under salt stress conditions. Bacterial two-hybrid analysis demonstrated that the AraC-type and NodD2 proteins can not only homodimerize but also form an AraC-type/NodD2 heterodimer, which could be responsible for the production of symbiotic molecules in the presence of osmotic stress conditions. In accordance with these findings, we renamed this AraC-type transcriptional regulator as OnfD (osmotic Nod factor regulator).

## RESULTS

### The OnfD amino acid sequence is conserved in *Rhizobium tropici* CIAT 899-related rhizobial strains but not in the other studied AraC-type regulators.

**FIG 1:**
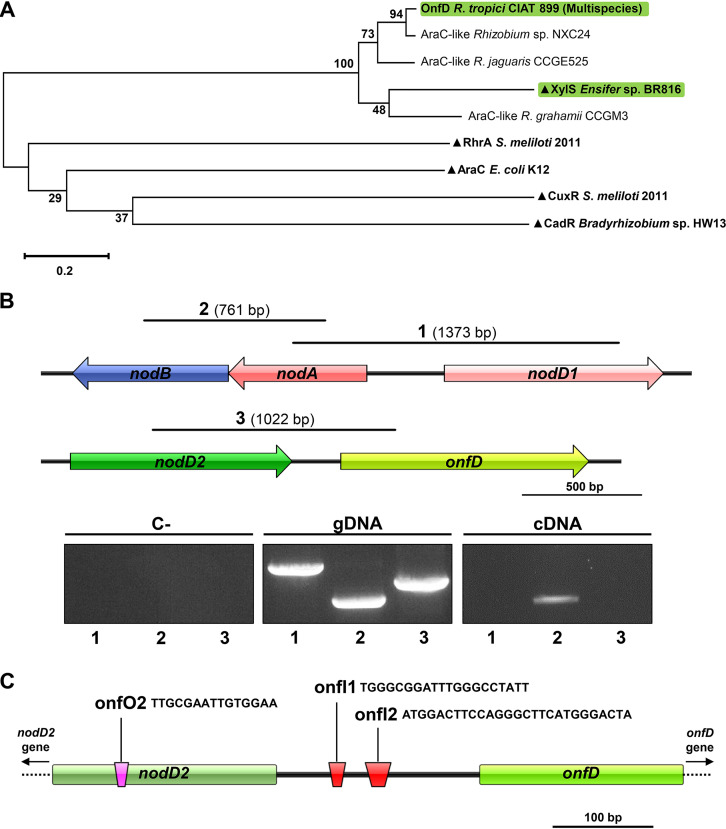
(A) Molecular phylogenetic tree analysis generated by using the maximum-likelihood method. The percentage of trees in which the associated taxa clustered together is shown next to the branches. The tree is drawn to scale, where the branch lengths are measured in the number of substitutions per site. All positions containing gaps and missing data were eliminated. AraC-type regulators (green) are involved in nodulation. ▴, AraC-type regulators with known functions. (B) Cotranscription assays of Rhizobium tropici CIAT 899 *nodD2* and *onfD* genes, including the genetic organization of the *nodD1-nodA1-nodB* and *nodD2-onfD* regions. Solid lines show the positions of the *nodD1-nodA1* (segment 1), *nodA1-nodB* (segment 2), and *nodD2-onfD* (segment 3) fragments that were assayed in PCR (gDNA) and RT-PCR (cDNA) experiments. Agarose gel electrophoresis shows the results from PCR and RT-PCR experiments. Amplicon 1 size, 1,373 bp; amplicon 2 size, 761 bp; amplicon 3 size, 1,022 bp. (C) Identification of the putative inducer (in red) and operator (in pink) sites of the *onfD* promoter region. Tandem repeat sequences are displayed.

OnfD (349 amino acids [aa], AGB73490.1), the deduced protein sequence of the CIAT 899 *onfD* gene with a predicted molecular mass of 42 kDa, is widespread in rhizobia and is highly conserved among CIAT 899-related strains. Its sequence is identical to the AraC-type regulators of *R. freirei* PRF 81 (ENN88367.1) and *Rhizobium* sp. strains CCGE531 (AYG70418.1), CCGE532 (AYG76959.1), and SEMIA 4088 (WP_004112928.1). It shows 94% identity to the *Rhizobium* sp. strain NXC24 protein (AVA24063.1), 81% identity to that of *R. jaguaris* CCGE525 (AYG64285.1), and <70% identity to an AraC-type regulator from *R. grahamii* CCGM3 (RDJ02018.1). As a first approach to understanding the putative function of OnfD, we performed a maximum-likelihood phylogeny analysis where we also included the AraC-type proteins characterized in previous studies (Fig. 1A). As expected, the AraC-type proteins of the strains phylogenetically related to CIAT 899 were grouped with OnfD. Interestingly, AraC/XylS (ORF816) from *Rhizobium* sp. strain BR816, which is involved in nodule formation, was also included in this group, suggesting that OnfD might be involved also in symbiosis. On the bottom branch of the tree, there were other AraC-like regulators (RhrA, CuxR, CadR, and AraC) that were not related phylogenetically either with OnfD or among themselves.

### The *nodD2* and *onfD* genes are not cotranscribed but are simultaneously activated in the presence of osmotic stress conditions.

The NB9 promoter in the CIAT 899 genome is located 979 and 2,123 bp upstream of the *nodD2* (RTCIAT899_PB01070) and *onfD* (RTCIAT899_PB01075) start codons, respectively. To determine the regulation of these two genes in the presence of flavonoids (3.7 μM apigenin) and osmotic stress (300 mM salt and 400 mM mannitol), RNA sequencing (RNA-seq) data deposited in public repositories (PRJNA470887, PRJNA326592, and PRJNA305690) were reanalyzed to obtain the fold change values for the *nodD2* and *onfD* genes with respect to noninduced cultures (Table 1). In the wild-type CIAT 899 strain, the transcriptional activation of *nodD2* and *onfD* was detected in cultures supplemented with salt (*nodD2 *=* *3.01 and *onfD *=* *3.87) or mannitol (*nodD2 *=* *2.26 and *onfD *=* *3.61) but not with apigenin (*nodD2* = −1.25 and *onfD *=* *1.15) and also not in a *nodD1* mutant background (Table 1). Nevertheless, in a *nodD2* mutant background, *onfD* expression in the presence of both osmotic stress conditions remained activated (salt = 2.55 and mannitol = 3.55), suggesting that NodD2 was not required for the *onfD* upregulation. To further investigate whether the two genes were cotranscribed, we used reverse transcription-PCR (RT-PCR) to detect putative CIAT 899 mRNAs covering the 3′ start site of *nodD2* and the 5′ end of *onfD* (Fig. 1B) that had been obtained in the absence of inducing molecules. As a positive control, we used primer pairs that allow the amplification of fragments of both *nodA1* and *nodB* genes. As a negative control, primer pairs of both *nodD1* and *nodA* genes (divergent orientation) were used. All of these primer pairs allowed the amplification of fragments of the expected size when CIAT 899 gDNA was used as the template. However, when cDNA from a noninduced culture of CIAT 899 was used, only the *nodA1-nodB* fragment (761 bp) was amplified (Fig. 1B). These results confirm that the *R. tropici* CIAT 899 *nodD2* and *onfD* genes do not belong to the same transcriptional unit.

**TABLE 1:** Fold change values of the *R. tropici* CIAT 899 genes located downstream of the *nod* box 9 in different genetic backgrounds

Locus tag/gene	Fold change^*a*^												
	CIAT 899 (12, 20, 21)			*nodD1* mutant (12, 20)			*nodD2* mutant (12, 20, 21)				*onfD* mutant (this study)		
	A	S	M	NIC	A	S	NIC	A	S	M	NIC	A	S
RTCIAT899_PB01570/*nodD2*	–1.25	3.01	2.26	–1.88	1.45	1.03	–	–	–	–	−1.10	1.10	−1.28
RTCIAT899_PB01575/*onfD*	1.15	3.87	3.61	–1.22	–1.29	1.44	1.27	1.49	2.55	3.51	–	–	–

a Significant transcriptional changes (≥3-fold induction respect to CIAT 899 noninduced cultures) in the presence of apigenin (3.7 μM) (A), salt (300 mM) (S), and mannitol (400 mM) (M) are highlighted in dark gray. NIC, noninduced culture. Slight transcriptional changes (≥2-fold induction/<3-fold induction) are highlighted in light gray. Source references are indicated in parentheses. –, gene is mutated on this genetic background.

The intergenic *nodD2-onfD* region (205 bp) was also investigated for the identification of direct (tandem) repeats, since a consensus sequence formed by tandem repeats is present in all AraC-type promoters, allowing protein binding (araI1 and araI2 inducer sites) (25). Two tandem repeats were identified in the promoter region of *onfD* (Fig. 1C), which could correspond to onfI1 and onfI2 inducer sites. As previously mentioned, the AraC regulator in E. coli acts as an activator or repressor depending on the presence of the ligand (24). Thus, in the absence of the inducing molecule, one monomer of the AraC occupies araI1 while the other monomer occupies the operator site araO2 (also a tandem repeat) ∼200 bp upstream (26–30). Interestingly, in CIAT 899, a tandem repeat was also identified 202 bp upstream of the putative onfI1 site, within the *nodD2* gene, which could correspond to the onfO2 site (Fig. 1C). This suggests that OnfD may be involved in the transcriptional regulation of the *onfD* and *nodD2* genes.

### OnfD activates *nod* gene expression and NF production under salt stress conditions.

**FIG 2:**
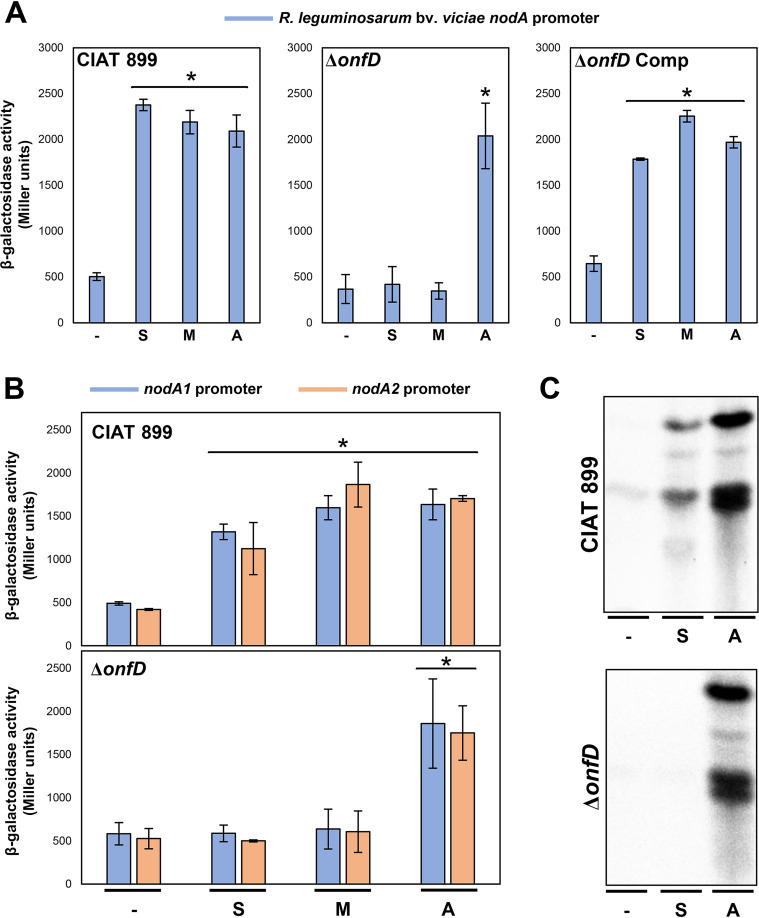
(A and B) β-Galactosidase activities of the CIAT 899 *ΔonfD* strain carrying plasmid pMP240 (R. leguminosarum bv. viciae *nodA* promoter) and pMP220, which contains the CIAT 899 *nodA1* and *nodA2* promoters fused to the *lacZ* gene. Assayed conditions included YM medium (–), YM supplemented with 300 mM NaCl (S), YM supplemented with 400 mM mannitol (M) and YM supplemented with 3.7 μM apigenin (A). Parameters were individually compared to that obtained in YM medium (–) by using a Mann-Whitney nonparametric test. Values tagged by asterisks (*) are significantly different at the level α = 5%. Error bars represent the standard deviations of data. (C) TLC analysis of Nod factors produced by CIAT 899 and *ΔonfD* mutant grown in YM medium (–), YM supplemented with 300 mM NaCl (S), and YM supplemented with 3.7 μM apigenin (A).

In a previous report from our research group, we determined that the CIAT 899 NodD2 protein regulates the production of NF under salt stress (21). However, the underlying molecular mechanism remains uncertain. Since both *nodD2* and *onfD*, located downstream of NB9, are activated under osmotic stress, we studied the putative implication of OnfD in *nod* gene upregulation and NF synthesis under these conditions. Thus, plasmid pMP240, harboring the conserved *nodA* promoter of R. leguminosarum bv. viciae fused to the *lacZ* reporter gene, was first transferred by conjugation to the CIAT 899 wild-type strain, as well as to the *onfD* deletion mutant (Δ*onfD*) and its complemented strain. β-Galactosidase assays showed similar activation levels in the presence of apigenin (3.7 μM), salt (300 mM), and mannitol (400 mM) in all the strains, with the exception of the *onfD* mutant, in which the activation of the promoter was detected in cultures supplemented with apigenin but not in the presence of salt or mannitol (Fig. 2A). This assay pointed out that OnfD is involved in the transcriptional activation of *nod* genes under osmotic stress conditions. To confirm the previous results, plasmids harboring the promoter regions containing the NB of the CIAT 899 *nodA1* and *nodA2* genes fused to *lacZ* were obtained and conjugated to the CIAT 899 wild-type strain and the *onfD* mutant. Then, β-galactosidase assays were performed in the presence or in the absence of apigenin, mannitol, and salt (Fig. 2B). As previously happened, *nodA1* and *nodA2* promoter activations were detected in both assayed strains in the presence of apigenin, whereas promoter activation under osmotic stress conditions was only detected in the wild-type strain (Fig. 2B). Considering the correlation between β-galactosidase assays and NF production (19–21) and in order to make more feasible the experimental approach, we only used 300 mM salt to induce osmotic stress. Thus, the correlation between upregulation of the *nodA1* and *nodA2* expression and synthesis of NF in the Δ*onfD* mutant was demonstrated by reversed-phase thin-layer chromatography (RP-TLC) (Fig. 2C). The NF profiles of the wild-type and the Δ*onfD* mutant strains were obtained radioactively labeling NF with ^14^C. The TLC assay showed that in the absence of OnfD, NF production was abolished in the presence of high concentrations of salt but not in the presence of apigenin. For an in-depth analysis, the chemical identification of the NF produced by the Δ*onfD* mutant was also obtained by UHPLC-MS/MS in the presence of apigenin (3.7 μM) or NaCl (300 mM) (see Table S1 in the supplemental material). In a previous study, 36 and 29 NF were detected in CIAT 899 supernatants in the presence of salt and apigenin, respectively (43). Here, as a positive control of NF production, the supernatant of the CIAT 899 wild-type strain grown in the presence of both apigenin and salt was employed (25 different NF). The mass spectrometry analyses showed that the Δ*onfD* strain synthesizes 29 NF in the presence of apigenin but only 4 NF under salt stress conditions. These results indicated that OnfD is involved in the production of NF in CIAT 899 under salt stress conditions.

**FIG 3:**
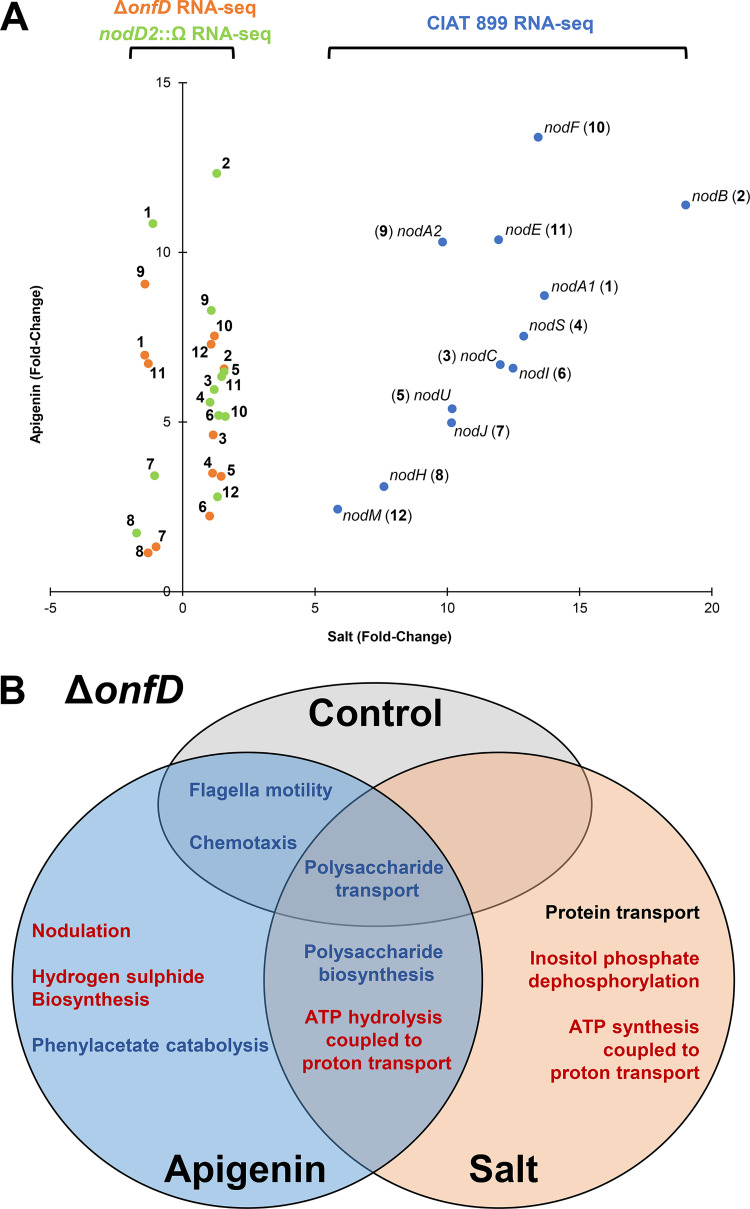
(A) Scatterplot representing the fold change values of the *nod* genes differentially expressed obtained in the RNA-seq assays from *R. tropici* CIAT 899 and *onfD* and *nodD2* mutants. Vertical and horizontal axis represent the values obtained in apigenin and salt conditions, respectively. Blue and light green circles represent the fold changes in our previous RNA-seq studies of the *R. tropici* CIAT 899 and *nodD2* mutant strains, respectively (12, 21). Orange circles represent the fold change levels observed in RNA-seq of the *onfD* mutant. (B) Venn diagram showing the biological processes differentially upregulated (in red), downregulated (in blue), or both (in black) in the *onfD* mutant in the presence or in the absence of apigenin and salt. The statistically overrepresented biological processes are from the data available at the UniProt database (GO) (see Data Set S3 in the supplemental material).

In our previous transcriptomic studies of CIAT 899, at least 17 genes located in the symbiotic plasmid and controlled by 5 different NB were overexpressed in the presence of both inducer molecules, apigenin and salt (12). To determine whether transcriptional activation of all the symbiosis-related genes under salt stress conditions is mediated by OnfD, 6 RNA-seq libraries (PRJNA605332) were generated from the *onfD* mutant grown in the presence or absence of apigenin (3.7 μM) and salt (NaCl 300 mM) (two independent biological experiments for each condition). These RNA-seq libraries were compared with an RNA-seq library from the wild-type strain grown in the absence of inducing molecules (PRJNA305690) (see Data Set S1 in the supplemental material). Quality control of each run, sample normalizations and statistical procedures were performed as previously described (12). The data set was validated by quantitative RT-PCR (qRT-PCR), obtaining in all cases positive correlation degrees in fold change values of 7 selected genes between the qRT-PCR and the RNA-seq data (see Data Set S2). Focusing our attention on these 17 symbiosis-related genes upregulated in the presence of both inducing molecules, RNA-seq experiments indicated that their transcriptional activation was detected in cultures of the Δ*onfD* mutant supplemented with apigenin but not with salt, confirming the role of OnfD in the transcriptional activation of these symbiotic genes under salt stress (Table 2). The fold change values of the *nod* genes in the *onfD* mutant in the presence of apigenin (induction) and salt (noninduction) were similar to those previously observed for the *nodD2* mutant strain under the same conditions (Fig. 3A). Interestingly, transcriptomic data from CIAT 899 grown in the presence of salt showed that the *nodD2* salt-mediated upregulation did not occur in the *onfD* mutant background, indicating that OnfD is involved in the regulation of *nodD2* expression under salt stress condition (Tables 1 and 2).

**TABLE 2:** Fold change values of the *R. tropici* CIAT 899 genes located downstream of functional *nod* boxes

NB	Locus tag/gene(s)	Function	Fold change^*a*^					
			CIAT 899 apigenin (12)	*nodD2* mutant apigenin (21)	*onfD* mutant apigenin (this study)	CIAT 899 salt (12)	*nodD2* mutant salt (21)	*onfD* mutant salt (this study)
1	RTCIAT899_PB1300 to RTCIAT899_PB01340/*nodABCSUIJH*	NF production	8.73 to 3.09	10.85 to 1.73	6.97 to 1.45	13.66 to 7.6	−1.12 to −1.74	−1.43 to −1.30
2	RTCIAT899_PB01095 to RTCIAT899_PB01110/*nodA2 hsnT nodFE*	NF production	10.3 to 10.37	8.29 to 6.34	9.07 to 6.73	9.81 to 11.94	1.07 to 1.46	−1.42 to −1.30
3	RTCIAT899_PB02710/*nodM*	NF production	2.43	2.79	7.29	5.85	1.31	1.08
4	RTCIAT899_PB00575 to RTCIAT899_PB00565/*y4wEF*	Synthesis of IAA	8.57 to 3.2	4.25 to −1.83	5.16 to 3.50	12.17 to 7.11	−1.12 to −1.3	−1.64 to −1.49
5	RTCIAT899_PB01550 to RTCIAT899_PB01545/HP	Unknown	6.75 to 4.26	4.1 to 2.35	2.46 to 1.91	28.65 to 14.37	1.96 to 1.42	1.14 to 1.04
9	RTCIAT899_PB01570/*nodD2*	Transcriptional regulation	−1.25		1.10	3.01		−1.28

a Transcriptional activation (≥3-fold induction with respect to CIAT 899 noninduced cultures) of several NB-controlled operons was demonstrated by RNA-seq experiments in the presence of both inducer molecules (dark gray boxes). Slight transcriptional changes (≥2-fold induction/<3-fold induction) are highlighted in light gray. HP, gene that codes for a hypothetical protein. Source references are indicated in parentheses.

The RNA-seq data also showed that other genes, related to nonsymbiotic biological processes, were differentially regulated in the *onfD* mutant. Therefore, a functional enrichment from the RNA-seq data in the *onfD* mutant was carried out to assign the statistically overrepresented biological processes (activated or repressed) using data available at the UniProt database (Gene Ontology [GO]). All the biological processes differentially regulated in the *onfD* mutant are shown in Data Set S3 in the supplemental material. Remarkably, in the absence of *nod* gene-inducing molecules, some of the differentially downregulated genes were involved in bacterial-type flagellum-dependent cell motility, chemotaxis and polysaccharide transport (Fig. 3B). All of these biological processes were also differentially activated/repressed in the presence of apigenin, indicating that OnfD may be involved in the regulation of several other biological processes. The presence of apigenin exclusively activated a set of biological processes such as hydrogen sulfide biosynthesis, phenylacetate catabolism, and, as expected, nodulation, whereas some biological processes were shared also with salt conditions, such as ATP hydrolysis, coupled proton transport, and polysaccharide synthesis and transport (this last one was downregulated in all tested conditions). Finally, the presence of salt specifically induced other biological processes such as ATP synthesis coupled to electron transport, inositol phosphate dephosphorylation, and protein transport.

### The activation of the OnfD-mediated NF production under salt stress is necessary for a successful symbiosis with *Phaseolus vulgaris*.

**FIG 4:**
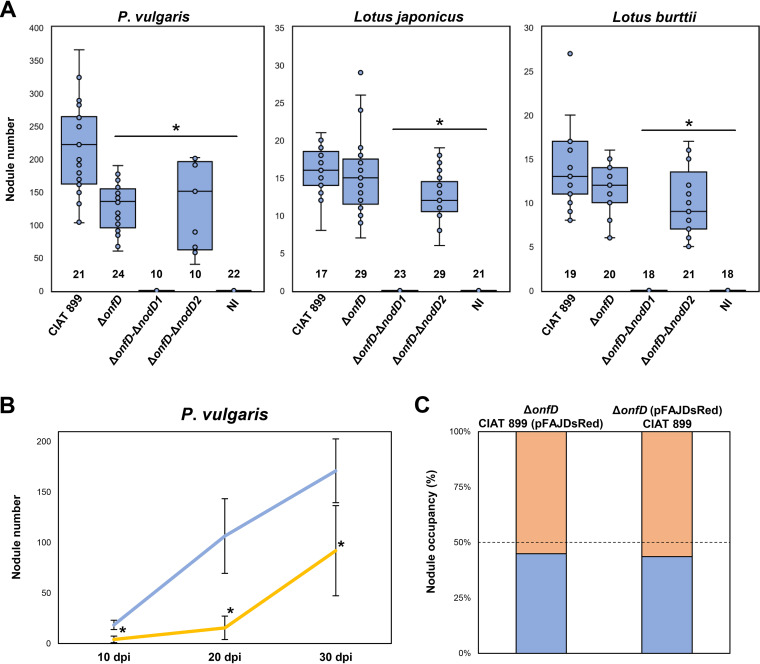
(A) Plant responses to inoculation of common bean and *Lotus* plants with different *R. tropici* CIAT 899 strains. P. vulgaris plants were evaluated 30 days postinfection (dpi). *Lotus* plants were evaluated 45 dpi. Parameters were individually compared to that obtained with the parental strain CIAT 899 by using the Mann-Whitney nonparametric test. Values tagged with an asterisk (*) are significantly different (α = 5%). The *n* value (number of plants assayed per strain) is indicated within bars. Error bars represent the standard deviations of data. Dots correspond to nodule number developed for each individual plant inoculated with different R. tropici CIAT 899 strains. (B) Nodulation kinetics of P. vulgaris inoculated with R. tropici CIAT 899 and its *onfD* mutant. Eight to sixteen plants were analyzed for determining nodule number per plant at each point of the time course experiments. The mutant nodule number (orange line) was individually compared to that obtained for the wild-type strain (blue line) by using a Mann-Whitney nonparametric test. Values tagged with asterisks (*) are significantly different at the level of α = 5%. Error bars represent the standard deviations of data. (C) Competition for nodulation assays of R. tropici CIAT 899andits *onfD* mutant derivative in P. vulgaris. Percentages of nodule occupancy were evaluated for both strains harboring (or not) the pFAJDsRed plasmid at 30 dpi.

To determine the symbiotic importance of the OnfD regulator, the symbiotic interactions of CIAT 899 and the *onfD* mutant on P. vulgaris, *L. japonicus*, and *L. burttii* were analyzed (Fig. 4A). In our previous study, the absence of NodD1 was enough to abolish nodulation in *L. japonicus*, while the absence of both NodD1 and NodD2 resulted in a lack of nodules in P. vulgaris and *L. burttii* roots (16, 21). In line with these previous results, we obtained two new CIAT 899 double-mutant strains for the *onfD-nodD1* and *onfD-nodD2* genes. The number of nodules in the two examined *Lotus* plants inoculated by the *onfD* mutant was not statistically different from those obtained in the parental strain CIAT 899, unless both NodD2/OnfD were absent, where significantly fewer nodules were observed. In contrast, both *onfD* and *onfD-nodD2* mutants presented a significantly reduced number of nodules in P. vulgaris in comparison to those plants inoculated with the parental strain. Interestingly, as occurred with the *nodD1-nodD2* mutant (21), no plants inoculated with *onfD-nodD1* presented nodules, highlighting the similar roles of NodD2 and OnfD in the symbiotic process. In line with these results, a time course nodulation assay in P. vulgaris showed an early delay in the nodulation process of the Δ*onfD* strain in comparison with the parental strain (Fig. 4B). However, the abilities of both strains for occupying common bean nodules were similar in the nodulation (see Fig. S1 in the supplemental material) and in the competition for nodulation assays (Fig. 4C). Overall, results obtained from nodulation tests indicate that the NF induced by the OnfD protein of CIAT 899 are required for nodule formation in P. vulgaris.

### OnfD forms dimers and interacts with NodD2 but not with NodD1.

**FIG 5:**
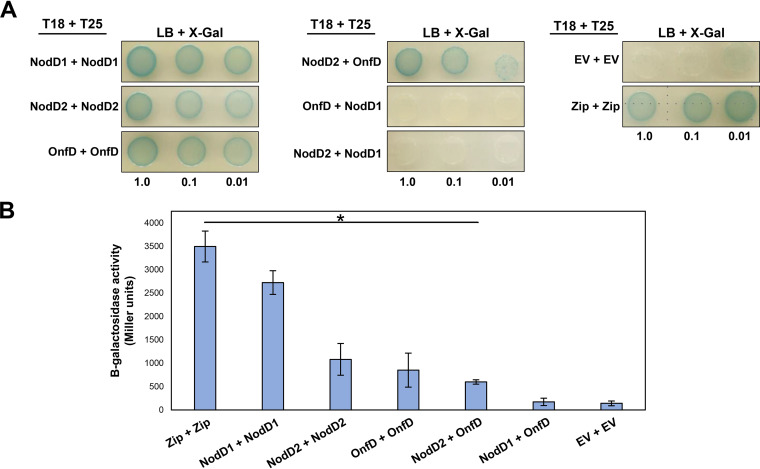
(A) Homo- and heterodimerization of Rhizobium tropici CIAT 899 NodD1, NodD2, and OnfD regulators. E. coli BTH101 (*cya*) was cotransformed with plasmids expressing the indicated T18 and T25 protein fusions. LacZ activity (blue) indicates protein-protein interaction. (B) Quantification of selected E. coli BTH101 (*cya*) cotransformants. The β-galactosidase activities were individually compared to that obtained with empty vector using a Mann-Whitney nonparametric test. Values tagged with asterisks (*) are significantly different at the level α = 5%. Zip, leucine zipper; EV, empty vector.

We investigated whether the NodD1, NodD2, and OnfD proteins were able to homo- and/or heterodimerize, a property required for the transcriptional activation ability of these regulators (26–28, 44). We used a bacterial two-hybrid system that depends on the reconstitution of adenylate cyclase activity, which is coupled with high β-galactosidase activity. The restoration of the adenylate cyclase activity is triggered by the interaction of two catalytic T25 and T18 subdomains in an adenyl cyclase deletion mutant background (E. coli *cya* strain) (45). After generating the fusion of our proteins of interest with the T25 and T18 subdomains, the results showed that NodD1, NodD2, and OnfD regulators form homodimers and/or homopolymers (tetramers, octamers, etc.) (Fig. 5A). As a positive control, we used the leucine zipper fused to the two subdomains of adenylate cyclases T25 and T18. The empty vectors harboring the T25 and T18 subdomains alone were used as negative controls of low adenylate cyclase activity levels. By using the same bacterial two-hybrid system, the interactions among NodD1, NodD2, and OnfD were also determined (Fig. 5A). Here, the OnfD-NodD2 two-hybrid combinations reconstitute adenylate cyclase activity, which indicates that both regulatory proteins may form a heterocomplex. In contrast, the interaction between NodD1-OnfD and NodD1-NodD2 was not detected. The formation of NodD2 and OnfD heterodimers was confirmed and quantified by β-galactosidase assays, displaying no significant differences among binding affinities regarding their respective homodimers (Fig. 5B). These results suggest that OnfD and NodD2 may be acting together during the upregulation of the *nod* genes under salt stress conditions.

### A structural model of OnfD predicts the presence of DNA-binding, ligand-binding, and dimerization domains.

To understand how OnfD acts during *nod* gene activation and NF synthesis under salt stress conditions, a prediction of the OnfD secondary structure on the basis of its amino acid sequence was determined (see Fig. S2 in the supplemental material). Surprisingly, the secondary structure of OnfD aligns with the structures of two phylogenetically distanced AraC-type proteins, CuxR protein from S. meliloti and ToxT from V. cholerae, despite their low amino acid sequence conservation (CuxR versus OnfD = 23.35% identity; ToxT versus OnfD = 27.59% identity) (see Fig. S2). We selected CuxR and ToxT because these proteins have known structures, determined by X-ray crystallography (ToxT PDB 3GBG and CuxR PDB 5NLA). We created two structural models of OnfD by using the CuxR and ToxT structures as references (see Fig. S3). In both OnfD models, we determined the presence of a putative DNA-binding (C-term) and a putative ligand-binding (N-term) domain composed of helix-turn-helix (HTH) and β-strands domains, respectively. These domains are typically present within the AraC family of regulators, and they may explain to some extent the role of OnfD in the activation of a different set of symbiotic genes under osmotic stress conditions. Moreover, the structural model of OnfD that used CuxR as a template showed one interesting finding: the presence of a hairpin domain composed of two α-helices that correspond with a dimerization domain of CuxR. This is another common feature of the AraC-type regulators that may explain the OnfD homodimerization ability observed in bacterial two-hybrid assays (Fig. 5).

## DISCUSSION

*R. tropici* CIAT 899 is one of the most important microsymbionts of P. vulgaris and is characterized by its ability to produce a large variety of NF not only in the presence of inducing flavonoid (3.7 μM apigenin) but also under osmotic stress conditions (300 mM salt and 400 mM mannitol) by means of the regulator proteins NodD1 and NodD2, respectively (15–17, 21, 22, 46–49). The activation patterns of the symbiotic genes are quite similar in the presence of both apigenin and salt since most of the CIAT 899 NB-controlled genes are activated at similar levels (12). An exception is found in genes located downstream of NB9, *nodD2* and RTCIAT899_PB01075 (coding for an AraC-type regulator, renamed as OnfD), which are only activated under salt stress conditions. The transcriptional activation of both genes is absent in a *nodD1* mutant background, suggesting that NodD1 is somehow required for the upregulation of both *nodD2* and *onfD* genes (21, 22). However, how salt is inducing the activation of the NodD2 protein and the mechanism by which NodD1 increases the total cytoplasmatic amount of both NodD2 and OnfD proteins remain unknown. According to results presented here, answers to these questions might be explained by the action of OnfD.

In our study, we have determined that OnfD is involved, like NodD2, in the transcriptional activation of the CIAT 899 symbiotic genes and in the synthesis of symbiotically active NF under salt stress conditions. First, we demonstrated that the activation of the CIAT 899 *nodA1* and *nodA2* promoters in the presence of salt or mannitol requires a functional OnfD, which correlates with the absence of NF overproduction under this stressing condition in the *onfD* mutant background (Fig. 2). Next, RNA-seq studies confirm the role of OnfD in the general activation of all previously defined CIAT 899 symbiotic genes in the presence of salt, since this upregulation was detected in the presence of apigenin but not with salt in the Δ*onfD* genetic background (Table 2 and Fig. 3). This regulatory role of OnfD is correlated with the presence of a putative DNA-binding domain in its structure, as shown in the structural model (see Fig. S3).

In the absence of OnfD, the activation patterns of these symbiotic genes were qualitatively and quantitatively similar to those previously reported for the *nodD2* mutant in the presence of salt and apigenin (21). This phenotypic parallelism between *onfD* and *nodD2* mutants is not restricted to NF production or transcriptomic profiles but is also evident for symbiotic behaviors: the absence of the OnfD protein was detrimental for nodulation with P. vulgaris but not with *Lotus* plants (Fig. 4A), in the same manner as for the *nodD2* mutant (16). This symbiotic phenotype observed in common bean seems to be the consequence of an early delay in the nodulation process of the *onfD* mutant (Fig. 4B) since competitiveness and nodule occupancy abilities are similar in both wild-type and mutant strains (Fig. 4C). However, identical behaviors in competition for nodulation assays might be the consequence of phenotypic complementation in the production of NF under osmotic stress conditions mediated by the wild-type strain. The role of another AraC-type regulator in symbiosis has also been briefly reported for *S. fredii* NGR234 since complementation with *orf816* and *nodD3* genes of *Rhizobium* sp. BR816 restored nodulation ability to a higher level compared to complementation with *nodD3* alone (42). Interestingly, the implication of OnfD and ORF816 in symbiosis also correlates with their proximity in our phylogenetic analysis (Fig. 1A), which might indicate a similar mechanism for both regulator proteins.

Results obtained in the bacterial two-hybrid assays are key to understanding how NodD2 and OnfD may be acting to activate the *nod* genes under osmotic stress conditions. Taking into consideration that OnfD forms homodimers and heterodimers with NodD2 and that AraC-type regulators play a dual role in controlling the expression target genes (as activators in the presence of ligand but as repressors in their absence) (26–32), we propose the following model of regulation of the *R. tropici* CIAT 899 *nodD2* and *onfD* genes and their role in the activation of the symbiotic genes under salt stress conditions (see Fig. S4 in the supplemental material). In the absence of salt, one monomer of the OnfD dimer would occupy the onfI1 site identified in the promoter region of *onfD*, while the other would occupy the onfO2 site located at the end of the previous ORF, the *nodD2* gene, leading to the formation of a DNA loop that would be blocking the basal transcriptional activation of the *onfD* and *nodD2* genes (see Fig. S4A). Under salt stress conditions, rhizobia accumulate intracellular compatible solutes such as sugars (sucrose, trehalose, maltose, cellobiose, turanose, gentiobiose, and palatinose), amino acids (mainly glutamate, glycine and proline), imino acids (pipecolate), ectoin, betaine, stachydrin, *N*-acetylglutaminylglutamine amide, and dimethylsulfoniopropionate (50). In fact, a study in *R. tropici* CIAT 899 demonstrated that this bacterium accumulates trehalose, mannitol and glutamate under salt stress (51). Therefore, one of these compatible solutes could act as a ligand and bind to OnfD, generating a conformational change that would lead to the occupancy of the inducer sites onfI1 and onfI2 and the induction of the *onfD* promoter (see Fig. S4B). Then, recruitment of the RNA polymerase by the dimeric and activated OnfD would be facilitated by the basal, but previously interrupted, transcription of the *nodD2* gene mediated by the NodD1 protein. As a consequence of this upregulation, the total cytoplasmatic amounts of both OnfD and NodD2 proteins would increase simultaneously in the presence of salt, allowing the formation of OnfD-NodD2 heterodimers. These heterodimers would bind to active NB, promoting the transcriptional activation of the *R. tropici* CIAT 899 symbiotic genes in the absence of inducing flavonoids. Thus, the lower rates of activation of these genes in the *nodD1* mutant with respect to the wild-type strain under salt conditions described in del Cerro et al. (21) could be explained by the low cytoplasmatic levels of NodD2 and OnfD. Our future efforts will be focused to validate (or deny) this model and to understand the implication of the osmotic stress-activated *nod* gene inductions during the symbiotic process.

In conclusion, we have demonstrated here that an AraC-type protein from a rhizobial strain, *R. tropici* CIAT 899, is directly involved in the transcriptional activation of the symbiotic genes and in the synthesis of symbiotically active NF under salt stress conditions. This mechanism seems to be widespread among rhizobia, since OnfD homologous regulators are present in strains phylogenetically related to CIAT 899 (Fig. 1). In addition to its role in nodulation, OnfD seems to be involved in the regulation of other biological processes, in the presence and absence of salt, which highlights the value of this regulator in some aspects of the *R. tropici* CIAT 899 way of life.

## MATERIALS AND METHODS

### Bacterial growth conditions, plasmids, and construction of mutants.

All strains and plasmids used in this work are listed in Table 3. In general, Escherichia coli strains were cultured on LB medium (52) at 37°C. E. coli BTH101 (*cya*) strains were grown at 30°C (45). *R. tropici* CIAT 899 strains were grown at 28°C on tryptone yeast extract (TY) medium (53), B– minimal medium (54) or yeast extract mannitol (YM) medium (55), supplemented when necessary with 3.7 μM apigenin, 300 mM NaCl, or 400 mM mannitol. When required, media were supplemented with the appropriate antibiotics as previously described (56). The Δ*onfD* and the double Δ*onfD* Δ*nodD1* and Δ*onfD* Δ*nodD2* deletion mutants were constructed by overlapping PCR extension, as previously described (57), where 765-, 819-, and 885-bp DNA fragments were deleted from *onfD*, *nodD1*, and *nodD2*, respectively. The deleted DNA fragments were first cloned into the pK18*mobsacB* vector, obtaining plasmids pK18*mobsacB*::Δ*onfD* (this study) and pK18*mobsacB*::Δ*nodD1* and pK18*mobsacB*::Δ*nodD2* (21). Then, the plasmids were transferred by conjugation and integrated in the genome of CIAT 899 by single recombination. Finally, a double homologue recombination event in which the wild-type copy of the gene, together with plasmid pK18mob*sacB*, was lost was selected. Mutated strains were confirmed by PCR and DNA-DNA hybridization (data not shown). For hybridization, DNA was blotted to Hybond-N nylon membranes (Amersham, Little Chalfont, UK), and the DigDNA method of Roche (Basel, Switzerland) was used according to the manufacturer’s instructions. In *cis* complementation of the *onfD* mutant was performed by integration of the plasmid pK18mob*sacB*::Δ*onfD* into the genome of CIAT 899, obtaining a derivative strain that harbors both the wild-type and the deletion mutant version of the *onfD* gene. Promoter regions (from the start codon to the stop codon of the previous gene) of the two different CIAT 899 *nodA* genes were amplified with specific primers (327 and 491 bp, respectively) and cloned into plasmid pMP220 upstream of the *lacZ* gene (58), obtaining plasmids pMP220::P_nodA1_ and pMP220::P_nodA2_. The CIAT 899 OnfD, NodD1, and NodD2 coding sequences were also cloned into pUT18, pUT18C, pKT25, and pKNT25 plasmids to fuse them to T18 and T25 subdomains in Bordetella pertussis *cya* at both the N-terminal and the C-terminal ends (45). Plasmid pFAJDsRed, which harbors a gene for red fluorescence, was also introduced in CIAT 899 and the Δ*onfD* derivative (59). All plasmids were transferred from E. coli to CIAT 899 by conjugation as previously described using plasmid pRK2013 as helper (60). Recombinant DNA techniques were performed according to the general protocols of Sambrook et al. (52). PCR amplifications were performed as previously described (61). Primers used in this study are listed in Table 4.

**TABLE 3:** Bacterial strains and plasmids used in this study

Strain or plasmid	Derivation and relevant properties	Source or reference
Strains		
*R. tropici*		
CIAT 899	Wild-type strain (Rif^r^)	17
*ΔonfD*	CIAT 899 *onfD* deletion mutant	This study
Δ*onfD* Δ*nodD1*	CIAT 899 Δ*onfD* Δ*nodD1* double deletion mutant	This study
Δ*onfD* Δ*nodD2*	CIAT 899 Δ*onfD* Δ*nodD2* double deletion mutant	This study
Δ*onfD* comp	CIAT 899 derivative strain that harbors both wild-type and deletion mutant versions of the *onfD* gene	This study
CIAT 899(pMP240)	*R. tropici* CIAT 899 carrying plasmid pMP240 (Tc^r^)	81
*ΔonfD*(pMP240)	CIAT 899 *onfD* mutant carrying plasmid pMP240 (Tc^r^)	This study
Δ*onfD* comp(pMP240)	CIAT 899 *onfD* complemented carrying plasmid pMP240 (Tc^r^)	This study
CIAT 899(pMP220::PnodA1)	CIAT 899 carrying plasmid pMP220::PnodA1 (Tc^r^)	This study
CIAT 899(pMP220::PnodA2)	CIAT 899 carrying plasmid pMP220::PnodA2 (Tc^r^)	This study
*ΔonfD*(pMP220::PnodA1)	CIAT 899 *onfD* mutant carrying plasmid pMP220::PnodA1 (Tc^r^)	This study
*ΔonfD*(pMP220::PnodA2)	CIAT 899 *onfD* mutant carrying plasmid pMP220::PnodA2 (Tc^r^)	This study
CIAT 899(pFAJDsRed)	CIAT 899 carrying plasmid pFAJDsRed (Tc^r^)	This study
*ΔonfD*(pFAJDsRed)	CIAT 899 *onfD* mutant carrying plasmid pFAJDsRed (Tc^r^)	This study
*E. coli*		
DH5α	*supE44 ΔlacU169 hsdR17 recA1 endA1 gyrA96 thi-1 relA1* (Nal^r^)	52
BTH101	F′ *cya‐99 araD139 galE15 galK16 rpsL1* (Str^r^) *hsdR2 mcrA1 mcrB1*	45
		
Plasmids		
pRK2013	Helper plasmid (Km^r^)	82
pMP220	Cloning vector (Tc^r^)	65
pMP220::PnodA1	pMP220 plasmid carrying the transcriptional fusion between the *R. tropici nodA1* promoter and the *lacZ* gene (Tc^r^)	This study
pMP220::PnodA2	pMP220 plasmid carrying the transcriptional fusion between the *R. tropici nodA2* promoter and the *lacZ* gene (Tc^r^)	This study
pMP220::PnodA3	pMP220 plasmid carrying the transcriptional fusion between the *R. tropici nodA3* promoter and the *lacZ* gene (Tc^r^)	This study
pMP240	pMP220 plasmid carrying the transcriptional fusion between the *R. leguminosarum* bv. viciae *nodA* promoter and the *lacZ* gene (Tc^r^)	65
pK18*mobsacB*	Cloning vector, suicide plasmid in rhizobia (Km^r^)	83
pK18*mobsacB*::Δ*onfD*	pK18*mobsacB* plasmid carrying the deleted version of the *onfD* gene (Km^r^)	This study
pK18*mobsacB*::Δ*nodD1*	pK18*mobsacB* plasmid carrying the deleted version of the *nodD1* gene (Km^r^)	21
pK18*mobsacB*::Δ*nodD2*	pK18*mobsacB* plasmid carrying the deleted version of the *nodD2* gene (Km^r^)	21
pUT18	*B. pertussis cya* T18‐expression plasmid (Km^r^)	45
pUT18C	*B. pertussis cya* T18C‐expression plasmid (Km^r^)	45
pKT25	*B. pertussis cya* T25‐expression plasmid (Km^r^)	45
pKNT25	*B. pertussis cya* NT25-expression plasmid (Km^r^)	45
pUT18C-zip	*B. pertussis cya* T18C‐leucine zipper fusion (Km^r^)	45
pKT25-zip	*B. pertussis cya* T25‐leucine zipper fusion (Km^r^)	45
pUT18::*nodD1*	*B. pertussis cya* T18‐NodD1 fusion (Km^r^)	This study
pUT18C::*nodD1*	*B. pertussis cya* T18C‐NodD1 fusion (Km^r^)	This study
pKT25::*nodD1*	*B. pertussis cya* T25‐NodD1 fusion (Km^r^)	This study
pKNT25::*nodD1*	*B. pertussis cya* NT25‐NodD1 fusion (Km^r^)	This study
pUT18C::*nodD2*	*B. pertussis cya* T18‐NodD2 fusion (Km^r^)	This study
pUT18::*nodD2*	*B. pertussis cya* T18C‐NodD2 fusion (Km^r^)	This study
pKT25::*nodD2*	*B. pertussis cya* T25‐NodD2 fusion (Km^r^)	This study
pKNT25::*nodD2*	*B. pertussis cya* NT25‐NodD2 fusion (Km^r^)	This study
pUT18C::*onfD*	*B. pertussis cya* T18‐OnfD fusion (Km^r^)	This study
pUT18::*onfD*	*B. pertussis cya* T18C‐OnfD fusion (Km^r^)	This study
pKT25::*onfD*	*B. pertussis cya* T25‐OnfD fusion (Km^r^)	This study
pKNT25::*onfD*	*B. pertussis cya* NT25‐OnfD fusion (Km^r^)	This study

**TABLE 4:** Primers used in this study

Primer	Nucleotide sequence (5′–3′)	Use
onfD-A	ATAGGATCCTGAACGTCTTTGAGTGGGTG	Mutagenesis
onfD-B	TCCCTCGTTGTATGCGCGGCTTTATACT	Mutagenesis
onfD-C	AGTATAAAGCCGCGCATACAACGAGGGA	Mutagenesis
onfD-D	AAATCTAGAAAGATGGTCATAGAGGAGGC	Mutagenesis
PnodA1-F	GAGGAATTCCTTTTCCTTCATTCAAGA	Cloning in pMP220 vector
PnodA1-R	TCTAGAGCAAAGACCTCCTTTTTC	Cloning in pMP220 vector
PnodA2-F	GAGGAATTCTTTTCGTCCTATCCATTT	Cloning in pMP220 vector
PnodA2-R	TCTAGAGCAAAGATCTCCTCTTCC	Cloning in pMP220 vector
PnodA3-F	AGAGAATTCACACTCTCTGCTCACCAT	Cloning in pMP220 vector
PnodA3-R	TCTAGATCAAAGACCTCTTCTTTT	Cloning in pMP220 vector
Hyb_onfD-F	AGAGGATCCCATGTTTCAAAGCAGCAGC	Cloning in two-hybrid vectors
Hyb_onfD-R	GAGGGTACCGCTCTGCGCAAGATTGAATG	Cloning in two-hybrid vectors
Hyb_nodD1-F	AGAGGATCCCATGCGCTTCAAAGGACTG	Cloning in two-hybrid vectors
Hyb_nodD1-R	GAGGGTACCGCATGCTCTGCCGACGGGAG	Cloning in two-hybrid vectors
Hyb_nodD2-R	AGAGGATCCCATGCGTTTCAAGGGTCTT	Cloning in two-hybrid vectors
Hyb_nodD2-R	GAGGGTACCGCAATGTCACTTTCGCGACA	Cloning in two-hybrid vectors
q_nodD1 F	CACGGTCGCTATGCGATTGGTA	qRT-PCR
q_nodD1 R	GTCGCGGCCAAATTCGGGAA	qRT-PCR
q_nodA2 F	GGGATTGTACGGGATACGCACCG	qRT-PCR
q_nodA2 R	CGTGCGGAAAAACCGTCACAGG	qRT-PCR
q_nodM F	TTGCAATAGCGTAGGCAAGC	qRT-PCR
q_nodM R	TGATGTCGCCTCCGAATTTC	qRT-PCR
q_nodB F	ACAAGGTCGCCAATCACA	qRT-PCR
q_nodB R	GCGCATATATTGCACCGA	qRT-PCR
q_y4wF F	CACCAGGCTGCTATGCGGAA	qRT-PCR
q_y4wF R	GGGTCTACCTGTGGCAATCCA	qRT-PCR
q_PB01550 F	TGAACTTGAAGGAAGCGCG	qRT-PCR
q_PB01550 R	TCGACGTGGCAAGGCATTAT	qRT-PCR
q_nodD2 F	AAAGCGTCTGGCAAGGGAAG	qRT-PCR
q_nodD2 R	TTTTCGTCGAACAGCTTCGC	qRT-PCR
q_16S F	ACACACGTGCTACAATGGTG	qRT-PCR
q_16S R	GCGATTACTAGCGATTCCAA	qRT-PCR
RT_nodD1 F	CACGGTCGCTATGCGATTGGTA	Cotranscription assays
RT_nodA1 R	ATGCCCGATCCCCAATCCCT	Cotranscription assays
RT_nodA1 F	TGATGCTAATGGTGTCGCGGC	Cotranscription assays
RT_nodB R	GCGCATATATTGCACCGA	Cotranscription assays
RT_nodD2 F	AAAGCGTCTGGCAAGGGAAG	Cotranscription assays
RT_onfD R	CGTTGATGGTAGGCTGGATT	Cotranscription assays

### Phylogenetic tree construction.

The phylogenetic tree was obtained using the software MEGA7 (62). Amino acid sequences of each AraC-type regulator were first aligned by MUSCLE (63). The evolutionary distances were computed using the maximum-likelihood method (64). All positions containing gaps and missing data were eliminated. The bootstrap value for this tree was 500.

### Determination of β-galactosidase activity.

To determinate the β-galactosidase activity, plasmid pMP240, which contains a transcriptional fusion between the R. leguminosarum bv. viciae *nodA* promoter and the *lacZ* gene, and the plasmids pMP220::P_nodA1_ and pMP220::P_nodA2_, which contain transcriptional fusions between the different CIAT 899 *nodA1* and *nodA2* promoters and the *lacZ* gene, were transferred by conjugation to the wild-type, mutant, and complemented strains (65). Assays of β-galactosidase activity were carried out as described by Zaat et al. (66). Units of β-galactosidase activity were calculated according to the method of Miller (67). The experiments were repeated three times with six replicates each time.

### RP-TLC analysis of NF.

RP-TLC analyses were performed according to Spaink et al. (68). Briefly, *R. tropici* CIAT 899 was grown on B– minimal medium, supplemented when necessary with inducing molecules. For the radiolabeling of Nod factors, 0.2 μCi of *N*-acetyl-d-[1-^14^C]-glucosamine (specific activity, 0.05 mCi) (Perkin-Elmer, Waltham, MA) was used. Cultures (1 ml) were grown to the end of the exponential growth phase, and the supernatant was extracted with water-saturated butanol. The butanol fraction was evaporated to dryness, and the resulting powder dissolved in 40 μl of water-saturated butanol. This solution (10 μl) was applied to the TLC plate (RP-18F254S; Merck, Darmstadt, Germany), where the Nod factors were separated with 50% acetonitrile/H_2_O (vol/vol) as the mobile phase. TLC plates were exposed to a Fuji BAS-IIIs film for 10 days, and the image was digitalized using a phosphorimage system (Fujifilm, Tokyo, Japan).

### Identification of NF.

The chemical identification of the NF was carried out by using an ultrahigh-pressure liquid chromatography system connected to a mass spectrometer (UHPLC-MS/MS) as previously described after growing the wild-type and the derivative mutant strains in B– minimal medium, supplemented when required with 300 mM NaCl or 3.7 μM apigenin (22).

### RNA and DNA extraction.

The *R. tropici* strains were grown at 28°C until stationary phase (optical density at 600 nm ≈ 1.2) on TY medium, supplemented when required with 300 mM NaCl or 3.7 μM apigenin. Total RNA was isolated using a High Pure RNA isolation kit (Roche, Basel, Switzerland) according to the manufacturer’s instructions. Verification of the amount and quality of total RNA samples was carried out using a Nanodrop 1000 spectrophotometer (Thermo Scientific, Waltham, MA) and a Qubit 2.0 fluorometer (Invitrogen, Carlsbad, CA). Two independent total RNA extractions were obtained for each condition. Genomic DNA (gDNA) was isolated using an i-genomic CTB DNA Extraction minikit (Intron, Gyeonggi-Do, South Korea) according to the manufacturer’s instructions.

### RNA sequencing, mapping of the RNA-seq data, assessment of differentially expressed genes, and functional categorization of genes.

rRNA was depleted using a MICROB Express bacterial mRNA purification kit (Ambion, Thermo Scientific), following the manufacturer’s protocol. The integrity and quality of the ribosome-depleted RNA were checked with an Agilent Bioanalyzer 2100 (Agilent Technologies, Santa Clara, CA). RNA sequencing was carried out by Sistemas Genómicos (Valencia, Spain) with the Illumina next-generation sequence (NGS) platform using the Illumina HiSeq 2000 sequencing instrument (Illumina, San Diego, California). Ribosome-depleted samples were used to generate whole-transcriptome libraries according to the manufacturer’s recommendations for sequencing on this NGS platform. Amplified cDNA quality was analyzed by the Bioanalyzer 2100 DNA 1000 kit (Agilent Technologies) and quantified by using a Qubit 2.0 fluorometer (Invitrogen, Thermo Scientific). The RNA-seq data discussed in this work are deposited in the Sequence Read Archive of NCBI under the accession numbers PRJNA605332 (this work), PRJNA470887, PRJNA326592, and PRJNA305690. The initial whole-transcriptome paired-end reads obtained from sequencing were mapped against the latest version of the *R. tropici* CIAT 899 genome deposited in NCBI (ASM33088v1) using the Life Technologies mapping algorithm version 1.3. Low-quality reads were eliminated using Picard Tools software version 1.83, retaining only high-quality reads. Gene prediction was estimated using the cufflinks method (69), and the expression levels were calculated using the HTSseq software, version 0.5.4p3 (70). This method eliminates multimapped reads, considering only unique reads for the gene expression estimation. The edge method version 3.2.4 was applied for differential expression analysis among conditions (71). This method uses a Poisson model to estimate the variance of the RNA-seq data for differential expression and relies on different normalized processes based on depth global samples, CG composition, and lengths of genes. Differential expression was established for those genes with a fold change lower or higher than −3 or 3, respectively, with a *P* value adjusted to 0.05. To assign statistically overrepresented functional categories in the presence of both *nod* gene-inducing molecules, an enrichment functional study was performed. Thus, genes were annotated using UniProt databases, and a hypergeometrical test using all genes as background and differential gene expression as the gene group of interest was applied (72). This statistical test calculates the statistical significance using *P* values (73), in this case evaluating the significance of functional categories. Those functional categories (biological processes) with a *P* value inferior to 0.1 (*P* adjusted) were considered overrepresented.

### Quantitative reverse transcription-PCR and cotranscription assays.

Results obtained in the RNA-seq analysis were validated by qRT-PCR of seven selected nodulation genes which represented differentially and non-differentially expressed genes in the presence of apigenin and salt. Total RNA was isolated using a High Pure RNA isolation kit (Roche), according to the manufacturer’s instructions. This (DNA-free) RNA was reverse transcribed into cDNA by using a PrimeScript RT reagent kit with gDNA Eraser (TaKaRa, Kusatsu, Japan). Quantitative PCR was performed using a LightCycler 480 (Roche) under the following conditions: 95°C for 10 min and then 40 cycles of 95°C for 30 s, 50°C for 30 s, and 72°C for 20 s, followed by the melting curve profile from 60 to 95°C to verify the specificity of the reaction. The *R. tropici* CIAT 899 16S rRNA gene was used as an internal control to normalize gene expression. The fold changes of two biological samples with three technical replicates of each condition were obtained using the ΔΔ*C_T_* method (74). Selected genes and primers are listed in Table 4.

To ensure that genomic DNA was not present in RNA samples in cotranscription assays, a control PCR was done employing primers showed in Table 4. Only those RNA samples showing no PCR amplification were selected for further work. Retrotranscription of total RNA was carried out using the PrimeScript RT reagent kit (TaKaRa), which includes a genomic DNA elimination step (gDNA Eraser) before RNA retrotranscription. PCR amplifications were performed as previously described (12). Primer pairs used for amplifying fragments of the *R. tropici* CIAT 899 *nodD2-onfD* region are shown in Table 4.

### Bacterial two-hybrid studies.

We used DNA sequences encoding OnfD, NodD1, and NodD2 to create fusions to the T18 and T25 catalytic subdomains of B. pertussis at both N-terminal and C-terminal ends. Bacterial two-hybrid studies were carried out as previously described (45). When appropriate monomers interact, the catalytic subdomains restore adenylate cyclase activity and cAMP synthesis, which were monitored by assaying a cAMP-regulated β-galactosidase reporter and confirmed by growth on M63 minimal medium (data not shown).

### Protein alignment and modeling.

The protein sequence and structure alignments were performed with PROMALS3D (75) and displayed with the Jalview software using the ClustalX coloring scheme (76). Models for OnfD were made with Phyre2 (77), a web server for protein modeling which uses homology detection methods to build three-dimensional models of proteins, using as a reference the structures of ToxT (PDB 3GBG) (78) and CuxR (PDB 5NLA) (38) proteins, previously determined by X-ray crystallography. Figures of the protein models were made with UCSF ChimeraX (79).

### Plant tests.

For the evaluation of the symbiotic phenotypes, the CIAT 899 strains were grown in YM medium until the concentration of 10^9^ cells ml^−1^. Surface-sterilized seeds of P. vulgaris, *L. burttii*, and *L. japonicus* were pregerminated and placed in sterilized jars (for P. vulgaris, Leonard jars) containing Farhäeus N-free solution as previously described (16). Growth conditions were 16 h at 26°C in the light and 8 h at 18°C in the dark, with 70% humidity. Nodulation parameters were evaluated after 30 days for P. vulgaris or 45 days for *Lotus* plants. Nodulation experiments were performed twice.

Kinetics of nodulation experiments were carried out as follows. Leonard jars containing two sterilized P. vulgaris seedlings were prepared and inoculated with 1 ml of 10^9^ cells ml^−1^ bacterial cultures. Plants inoculated with CIAT 899 parental and *onfD* mutant strains were grown in a plant growth chamber with a 16 h photoperiod at 25°C (light) and 18°C (darkness). Nodulation of eight plants was scored at 10, 20, and 30 days after inoculation. At least two independent experiments were carried out for each strain.

Competition for nodulation experiments on P. vulgaris were performed using wild-type and mutant strains harboring (or not) the pFAJDsRed plasmid, which provides red color to colonies (59). These bacteria were grown to 10^9^ cells ml^−1^, and four to five Leonard jar assemblies containing two P. vulgaris seedlings each were inoculated with 1 ml of a mixture of bacterial competitors in a 1:1 ratio. Plants were grown for 4 weeks in a plant growth chamber under the growth conditions described above. To identify bacteria occupying the nodules, bean nodules were surface sterilized by immersing them in bleach (1/5 dilution of a stock solution containing 14% [wt/vol] sodium hypochlorite) for 2 min, followed by five washing steps in sterilized distilled water. The effectiveness of the surface-sterilizing treatment was checked by inoculating TY plates with 20-μl aliquots from the last washing step. Individual surface-sterilized nodules were crushed in 30 μl of sterilized distilled water, and 20-μl aliquots were used to inoculate TY plates. Nodule occupancy was determined by assessing the ratio of red-colored nodule isolates. At least 10 colonies from each isolate were analyzed in order to check the possibility of nodules containing both inoculants.

For nodule occupancy visualization, 30-day-old *P. vulgaris* nodules formed in plants inoculated with CIAT 899 and CIAT Δ*onfD* strains carrying the DsRed fluorescent marker were embedded in 6% agarose in water and sliced in thick layer sections (30 μm) using a Leica VT 1000S vibratome (Wetzlar, Germany). Sections of nodules were stained with 0.04% calcofluor and observed by using a Zeiss ApoTome.2 (Jena, Germany) fluorescence microscope as previously described (80).

### Data availability.

Newly determined RNA-seq data in this study were deposited in the NCBI Sequence Read Archive under accession number PRJNA605332. Other RNA-seq data used here can be found under accession numbers PRJNA470887, PRJNA326592, and PRJNA305690.

## Supplementary Material

Supplemental file 1

Supplemental file 2

Supplemental file 3

Supplemental file 4
