# Supplementary material for: OnfD, an AraC-Type Transcriptional Regulator Encoded by Rhizobium tropici CIAT 899 and Involved in Nod Factor Synthesis and Symbiosis
Source: Appl Environ Microbiol. 2020 Sep 17;86(19):e01297-20. doi: 10.1128/AEM.01297-20 (PMC7499043; doi:10.1128/AEM.01297-20)
Supplement: Supplemental file 1 [file AEM.01297-20-s0001.pdf]

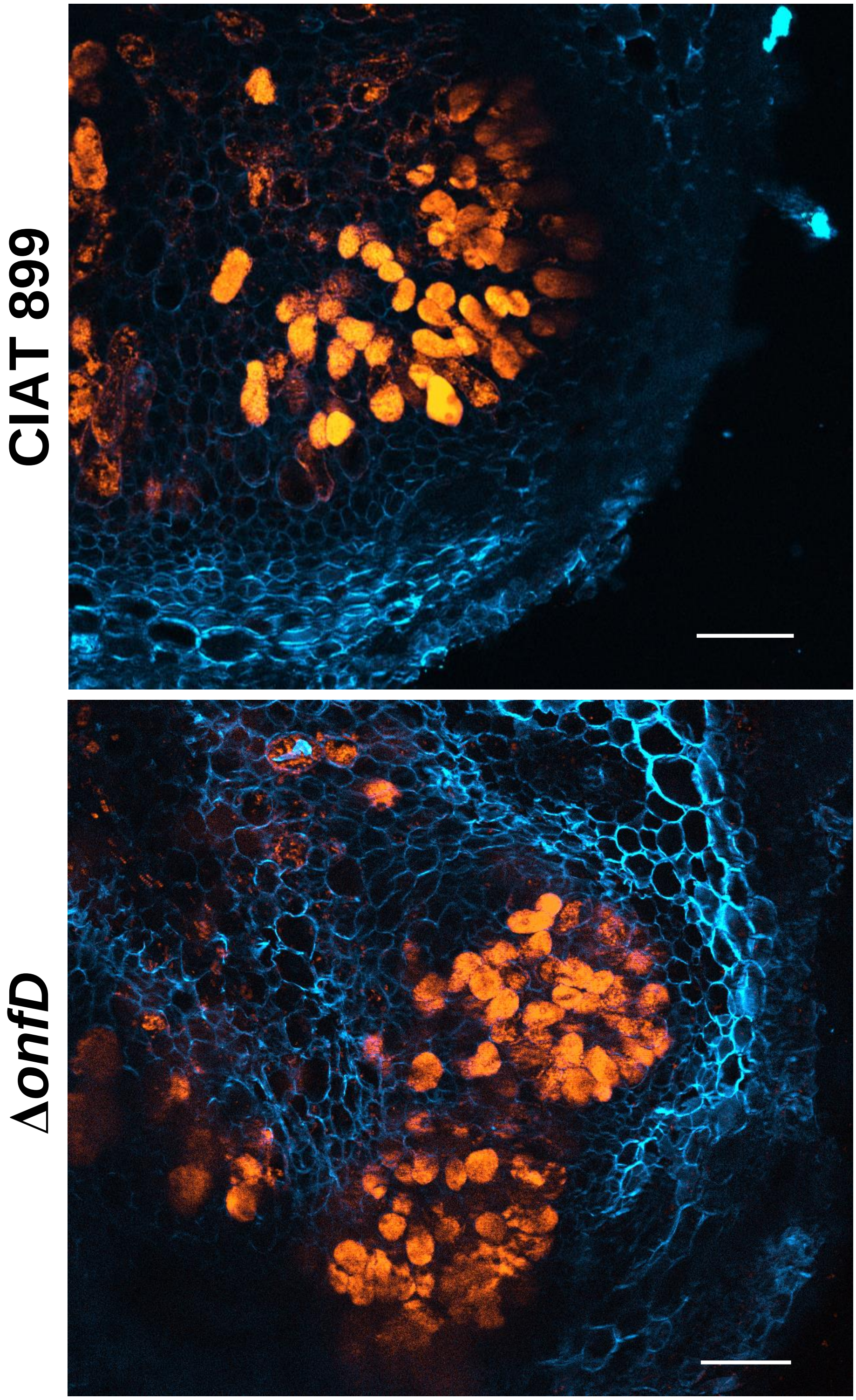

Fig S1. Nodule occupancy of *Rhizobium tropici* CIAT 899 and the *onfD* mutant in *P. vulgaris*. Light microscopy images of nodules of *P. vulgaris* were captured from plants inoculated with the abovementioned strains carrying the DsRed reporter gene. Bars correspond to 100 µm. Images were captured at 30 dpi.

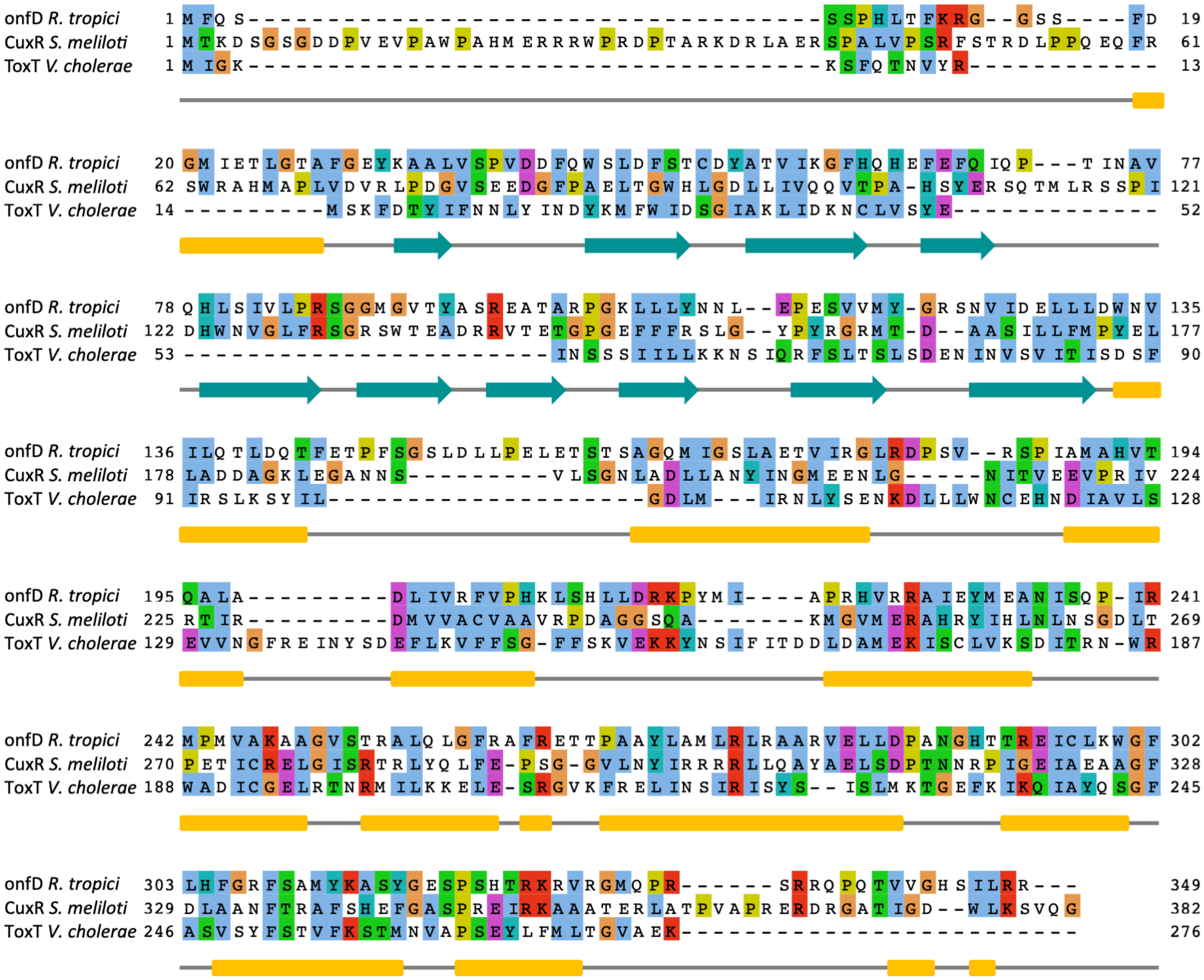

Fig. S2. Sequence alignment of OnfD, CuxR and ToxT. The consensus secondary structure motifs (α-helices as yellow tubes, β-strands as cyan arrows) are shown underneath the alignment. The protein sequence and structure alignments were performed with PROMALS3D and displayed with the program Jalview using the ClustalX coloring scheme.

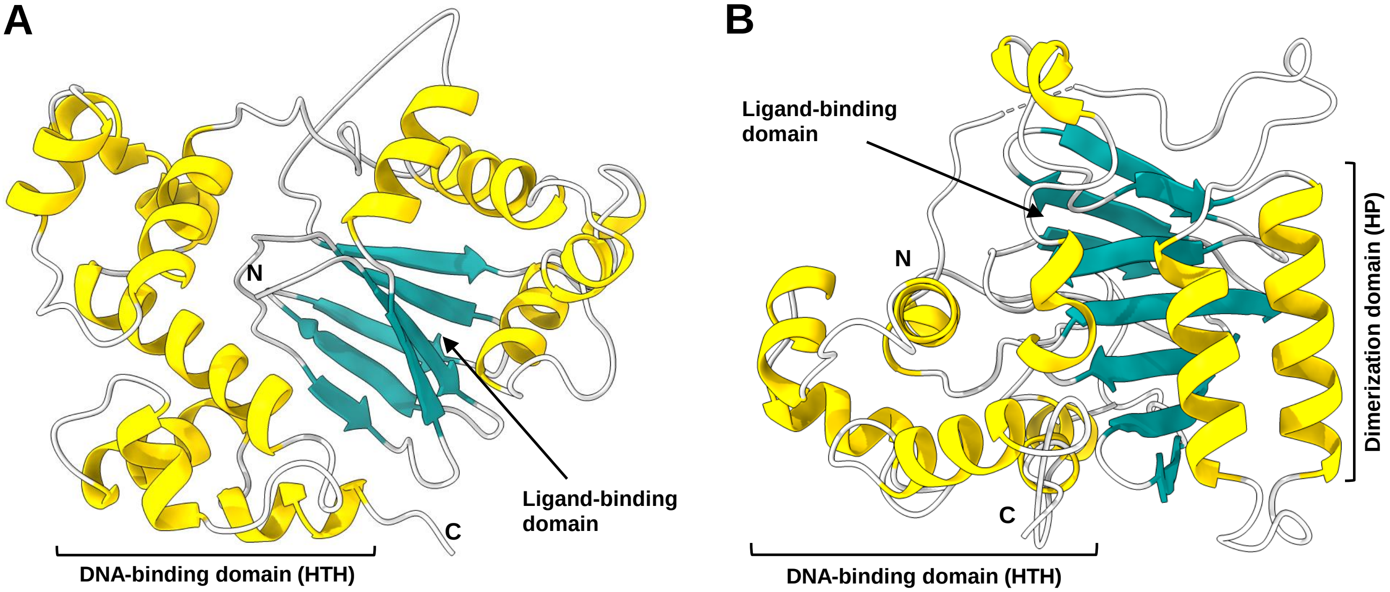

Fig S3. Structural models of OnfD based on the crystal structures of *V. cholerae* ToxT (3GBG, **A**) and *S. meliloti* CuxR (5NLA, **B**). Ribbon diagrams of the OnfD protein models show α-helices (yellow), β-strands (cyan), and loops (grey). The N and C termini (N and C, respectively) are labelled. HTH = Helix-turn-helix, HP = Helical hairpin.

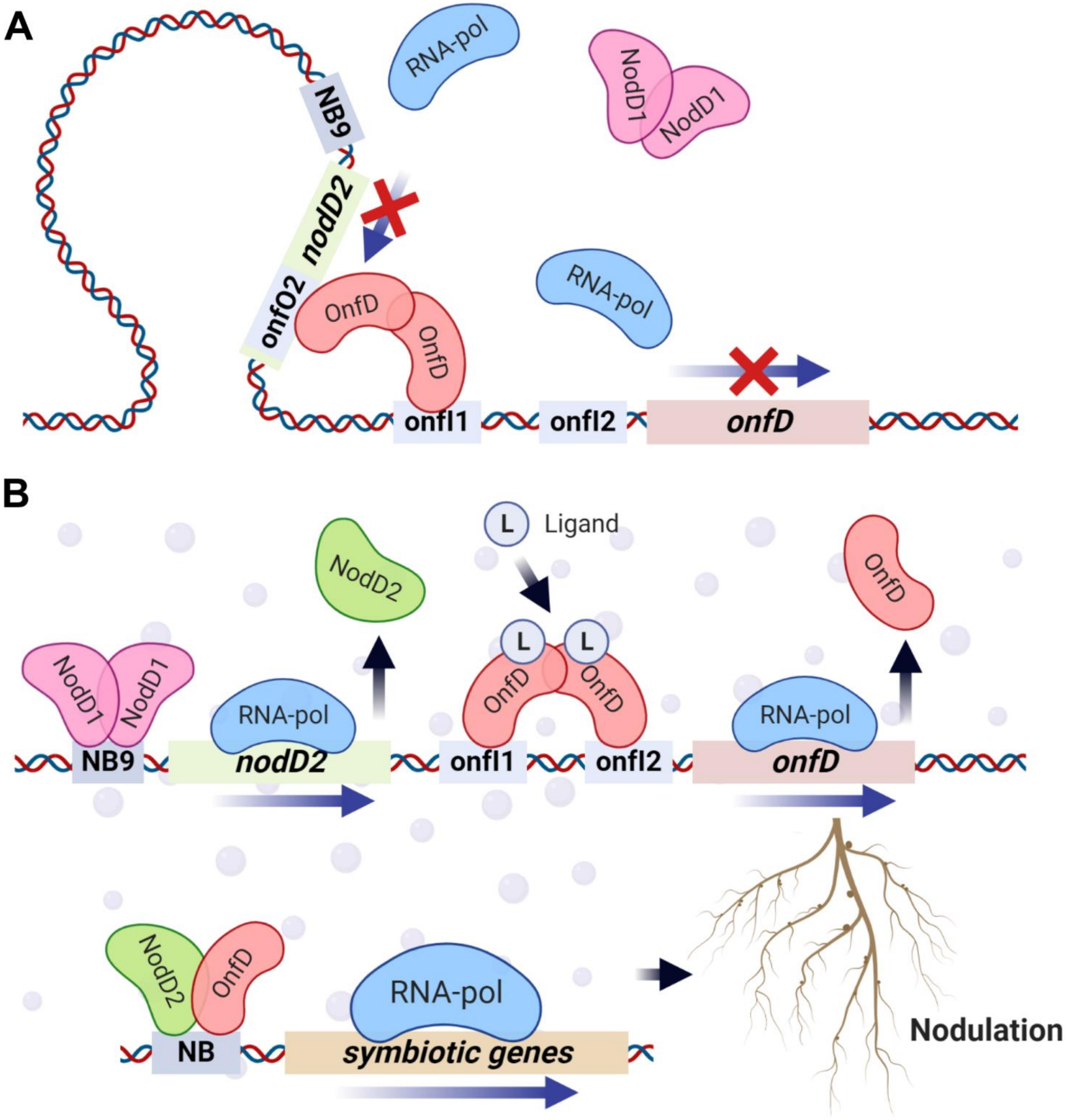

Fig. S4. Model of regulation of the *Rhizobium tropici* CIAT 899 *nodD2* and *onfD* genes in the presence or absence of salt. **A**. In the absence of salt, each monomer of the OnfD dimer would occupy the onfI1 and onfO2 site, leading to the formation of a DNA loop that would be blocking the up-regulation of the *nodD2* and *onfD* gene. **B**. Under salt stressing conditions, one of the intracellular compatible solutes (Ligand, L) would bind to the dimeric OnfD, generating a conformational change that would lead the occupancy of the inducer sites onfI1 and onfI2. Recruitment of the RNA polymerase by the dimeric and activated OnfD would be facilitated for the basal transcription of the *nodD2* gene mediated by NodD1. As a consequence, OnfD and NodD2 cytoplasmatic amounts increase, allowing the formation of OnfD-NodD2 heterodimers, which would interact to all NB activated in the presence of salt (NB1-5) promoting the transcriptional activation of the CIAT 899 symbiotic genes. Figure created with BioRender.com.

Table S1. NF synthetized in the absence and in the presence of apigenin (3.7 µM) or salt (300 mM) by the *onfD* mutant.

NF structure[a]	CIAT 899 + apigenin and salt[b]	Δ*onfD*[b]	Δ*onfD* + salt[b]	Δ*onfD* + apigenin[b]
**III ($C_{16:0}$)**	-	-	-	+
**III ($C_{16:0}$, NMe)**	-	-	-	+
**III ($C_{18:0}$)**	-	-	-	+
**III ($C_{18:1}$)**	-	-	-	+
**IV ($C_{14:0}$)**	-	-	-	+
**IV ($C_{14:0}$, NMe)**	-	-	-	+
**IV ($C_{16:0}$)**	+	-	-	+
**IV ($C_{16:0}$, NMe)**	-	-	-	+
**IV ($C_{16:1}$)**	+	-	-	+
**IV ($C_{18:0}$)**	+	-	-	+
**IV ($C_{18:0}$, NMe)**	+	-	-	+
**IV ($C_{18:1}$)**	+	-	+	+
**IV ($C_{18:1}$)dNAc**	-	-	-	+
**IV ($C_{18:1}$, NMe)**	+	-	-	+
**IV ($C_{18:1}$, NMe, S)**	-	-	-	+
**IV ($C_{18:1}$, S)**	-	-	-	+
**IV ($C_{20:0}$)**	+	-	-	-
**V ($C_{14:0}$)**	+	-	-	+
**V ($C_{14:0}$, NMe)**	+	-	-	+
**V ($C_{16:0}$) dNAc**	+	-	-	-
**V ($C_{16:0}$, NMe)**	-	-	-	+
**V ($C_{16:0}$, NMe)**	+	-	-	-
**V ($C_{16:0}$, NMe, S)**	+	-	-	-
**V ($C_{16:1}$, NMe)**	+	-	-	+
**V ($C_{18:0}$)**	+	-	+	+
**V ($C_{18:0}$, NMe)**	+	-	-	+
**V ($C_{18:0}$, NMe, S)**	+	-	-	+
**V ($C_{18:0}$, S)**	+	-	-	-
**V ($C_{18:1}$)**	+	+	+	+
**V ($C_{18:1}$) dNAc**	-	-	-	+
**V ($C_{18:1}$, NMe)**	-	-	-	+
**V ($C_{18:1}$, NMe) dNAc**	-	-	-	+
**V ($C_{18:1}$, NMe, S)**	+	-	-	+
**V ($C_{18:1}$, S)**	+	-	+	+
**V ($C_{20:0}$)**	+	-	-	-
**V ($C_{20:0}$, NMe)**	+	-	-	-
**V ($C_{20:0}$, NMe, S)**	+	-	-	-
**V ($C_{20:1}$, NMe, S)**	+	-	-	-
**V ($C_{20:1}$, NMe)**	+	-	-	-
**Number of LCO synthetized**	**25**	**1**	**4**	**29**

[a] NF structure are represented following the convention (Spaink HP, Okker RJ, Wijffelman CA, Pees E, Lugtenberg BJ. 1989. Promoters in the nodulation region of the *Rhizobium leguminosarum* Sym plasmid pRL1JI. Plant Mol Biol 9:27-39) that indicates the number of GlcNAc residues in the backbone (Roman numeral), the length and degree of unsatured of the fatty acyl chain, and the other substituents, which are listed in the order in which they appear, moving clockwise from the fatty acid. NMe, N-methyl group at the glucosamine non-reducing residue; S, sulfate at reducing glucosamine residue; dNAc in 3 or 4, deacetylated at glucosamine residues number 3 or 4; OH, hydroxylated fatty acid. [b]Symbol: +, detected; -, non-detected.
